# Structural basis of Δ^9^-THC analog activity at the Cannabinoid 1 receptor

**DOI:** 10.21203/rs.3.rs-4277209/v1

**Published:** 2024-05-21

**Authors:** David Gloriam, Thor Thorsen, Yashraj Kulkarni, David Sykes, Andreas Bøggild, Taner Drace, Pattarin Hompluem, Christos Iliopoulos-Tsoutsouvas, Spyros Nikas, Henrik Daver, Alexandros Makriyannis, Poul Nissen, Michael Gajhede, Dmitry Veprintsev, Thomas Boesen, Jette Kastrup

**Affiliations:** University of Copenhagen; University of Copenhagen; University of Copenhagen; University of Nottingham; Aarhus University; Aarhus University; University of Nottingham; Northeastern University; Northeastern University; University of Copenhagen; Northeastern University; Aarhus University; University of Copenhagen; University of Nottingham; Aarhus University; University of Copenhagen

## Abstract

Δ^9^-tetrahydrocannabinol (THC) is the principal psychoactive compound derived from the cannabis plant Cannabis sativa and approved for emetic conditions, appetite stimulation and sleep apnea relief. THC’s psychoactive actions are mediated primarily by the cannabinoid receptor CB_1_. Here, we determine the cryo-EM structure of HU210, a THC analog and widely used tool compound, bound to CB_1_ and its primary transducer, G_i1_. We leverage this structure for docking and 1,000 ns molecular dynamics simulations of THC and 10 structural analogs delineating their spatiotemporal interactions at the molecular level. Furthermore, we pharmacologically profile their recruitment of G_i_ and β-arrestins and reversibility of binding from an active complex. By combining detailed CB_1_ structural information with molecular models and signaling data we uncover the differential spatiotemporal interactions these ligands make to receptors governing potency, efficacy, bias and kinetics. This may help explain the actions of abused substances, advance fundamental receptor activation studies and design better medicines.

## Introduction

Δ^9^-tetrahydrocannabinol (THC) is the principal psychoactive compound derived from the cannabis plant, Cannabis sativa. The beneficial physiological effects of THC include analgesia and appetite stimulation^1^, while harmful effects include e.g., paranoia and anxiety^2^. As a marketed pharmaceutical Dronabinol, THC is approved as an antiemetic, appetite stimulant and reliever of sleep apnea^3^. The THC analog Cannabidiol is used for managing anxiety, insomnia, paediatric epilepsy and chronic pain, and these two drugs are combined in Nabiximols, treating neuropathic pain and spasticity in multiple sclerosis. A synthetic THC analog, Nabilone, is also available as an antiemetic and adjunct analgesic for neuropathic pain effective in relieving fibromyalgia^4^ and multiple sclerosis^5^. Clinical trials are investigating cannabinoids for treating glioblastoma and a variety of neurological disorders, including Parkinson’s disease, Alzheimer’s disease, Huntington’s disease, amyotrophic lateral sclerosis, traumatic brain injury and stroke^6^.

The main targets of THC are the cannabinoid receptors, CB_1_ and CB_2_. Psychoactive effects are mediated by CB_1_ in the cerebral cortex, cerebellum, and basal ganglia, whereas CB_2_ is mainly expressed in cells of the immune system^7^. CB_1_ mediates its signaling primarily by G_i/o_ that inhibits adenylate cyclase which in turn decreases the cellular concentration of cyclic AMP^8^. In addition, CB_1_ also couples to G_s_, G_q/11_ and arrestins^9–11^. Several CB_1_ ligands exhibit pathway-biased signaling^12,13^ i.e., preferential activation of a specific transducer pathway. Biased signaling can lead to functionally selective responses opening for the design of drugs with more beneficial therapeutic profiles and fewer side effects^14,15^. The multiple pathways, biased ligands and clinical indications make CB_1_ an appealing system for studying biased signaling.

The structural coverage of complexes of THC analogs and cannabinoid receptors presently spans AM841^16,17^, CP55940^18–20^ and AM11542^16^ bound to CB_1_ and AM12033^17^, CP55940^21^ and HU243^21^ bound to CB_2_. The resolution of the structures spans 2.8–3.4 Å and three structures are fully activated cryo-EM complexes with G_i1-2_, whereas the AM11542-CB_1_ complex is a crystal structure with a fusion protein, flavodoxin, that restrains TM6 in an active state. The structure coordinates’ coverage of the overall CB_1_ protein sequence range between 58 and 62% of residues among all active-state structures of this receptor (Extended Data Table 1) due to the intrinsic flexibility of the N-/C-termini and long third intracellular loop (ICL3).

THC has a tricyclic core consisting of phenyl, pyran and cyclohexene rings that are indexed A, B and C, respectively (Extended Data Fig. 1). Opening of the pyran ring leads to compounds referred to as Cannabidiols that have weaker affinity to CB_1–2_ and psychoactivity^22^ than the THC class. Numerous THC analogs have been published, whereof HU210 is among the most potent tool compounds. HU210 is 100–800 times more potent than naturally sourced THC in discrimination tests in animal studies^23^ and has a binding affinity at human CB_1_ that is ~700 higher than for THC^24^. HU243 is a close structural analog of HU210, differing just by the lack of a double bond in C8-C9 of the C ring, sharing its high potency^24^. Additional potent synthetic THC analogs include the widely used tool compound, CP55940 as well as AM841 and AM11542. In contrast, the phytocannabinoid Cannabinol^25^ and synthetic ligands L759633^26^ and JWH133^27^ have lower affinities and potencies at CB_1_ and less strong psychoactive effects than THC. Inverse agonism is observed for Tetrahydrocannabivarin (THCv) that has the opposite effect of THC by suppressing appetite and controlling blood glucose levels^28^, making it a potential treatment for obesity and diabetes.

Structure-activity relationship studies of Δ^9^-THC and Δ^8^-THC have focused mainly on the three major pharmacophore features: *i)* the phenolic hydroxyl group at the C1 position on the A-ring, *ii)* the methyl group at the C9 position on the C-ring, and *iii*) the alkyl side chain at the C3 position on the A-ring^29–31^ (Extended Data Fig. 1). C1 phenolic hydroxyl removal (JWH133) and etherification (L759633) increase selectivity for CB_2_ over CB_1_ activation by 153-fold^27^ and 200-fold^26^, respectively. Substitution of the C9 methyl with hydroxyl (CP55940) or carbonyl (Nabilone) groups improves affinity, but not selectivity^31^. A shorter and longer alkyl tail compared to THC decreases and increases binding affinity and potency, respectively,^32–34^ and five to eight carbons are considered optimal^32,35^. Elongation with large halogens at the terminal position (as in AM11542) increases binding affinity. Binding affinity can also be enhanced by methyl substitution at the C1’ and C2’ positions^31^. Here, 1’,2’-substitution gives as good binding affinity but 1’,1’-dimethyl substituted compounds are easier to synthesize. Hitherto, these and other structure-activity studies have predominantly focused on analog substitutions correlated to binding activity or selectivity, and assessment of potency has typically addressed effective dose *in vivo*^29–31^. This leaves a need for more molecular mechanistic studies covering ligand-receptor interactions and dissecting additional pharmacological parameters under consistent experimental conditions.

Here, we benchmark the potency and efficacy of THC and 10 analogs for the recruitment of the primary transducer family, G_i/o_ and b-arrestin. Furthermore, we solve the cryo-EM structure of the HU210/CB_1_/G_i1_ signaling complex and leverage this template to obtain consistent ligand binding modes and spatiotemporal interactions through 1,000 ns molecular dynamics (MD) simulations. Combining these approaches, we expand the structural rationale of THC analogs beyond ligand moieties to dynamic receptor interactions delineating determinants for potency, efficacy, kinetics and biased signaling.

## Results

### Cryo-EM structure of the HU210/CB_1_/G_i1_ signaling complex

**Figure 1:**
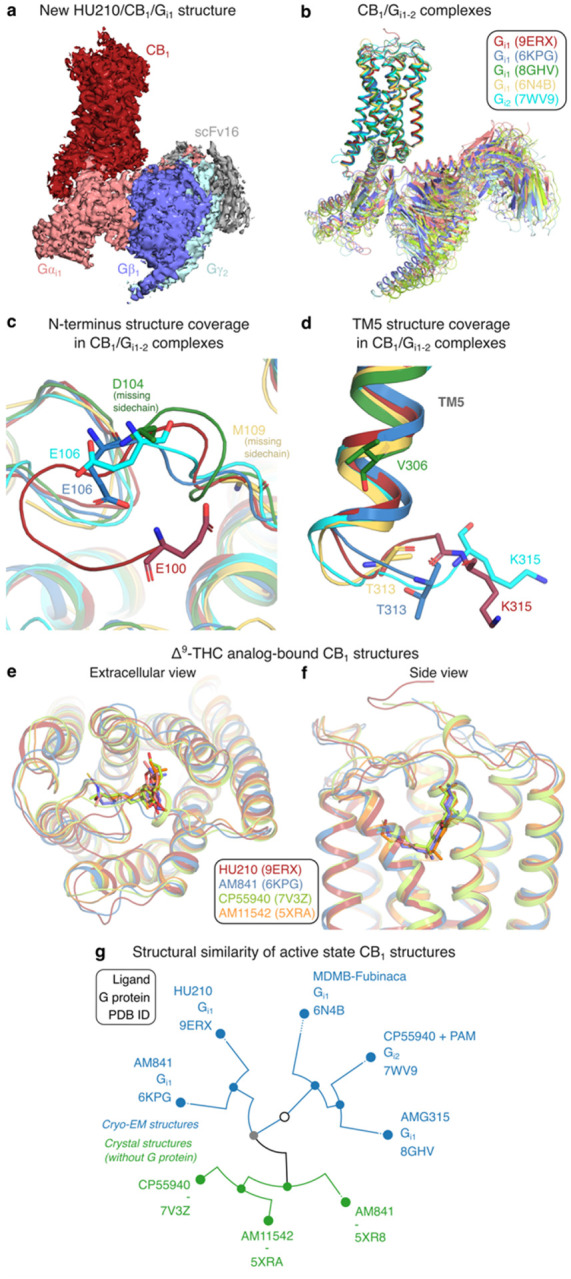
Active state CB_1_ structures – completeness and complexes with G_i_ and THC analogs. **a,** Cryo-EM density map (sharpened) of the HU-210/CB_1_/G_i1_ signaling complex. **b,** CB_1_-G_i_ complexes^17,19,38,39^ superposed based on the seven transmembrane helix backbone atoms. **c, d,** N-terminal (**c**) and TM5 (**d**) segments uniquely covered by the HU-210/CB_1_/G_i1_ structure. The shown residues are the terminal residue in each structure. **e, f,** Extracellular (**e**) and (**f**) side views of THC analog/CB_1_ structure complexes. **g,** Structure similarity tree of active state CB_1_ structures based on a conformational clustering in GPCRdb making a superposition-independent all-to-all residue distance comparisons across the transmembrane domain^74^.

The human CB_1_ receptor (residues 2–472) and a G_i1_ double mutant (G203A, A326S) with increased propensity to form complex with the receptor^36,37^ were co-expressed in Sf9 insect cells. A stable HU210/CB_1_/G_i1_ complex was formed in cell membranes by binding of the ScFv16 fragment antibody (Fab), and hydrolysis of released GDP nucleotides using Apyrase enzyme. The complex was purified in LMNG detergent micelles, using a M1 anti-Flag affinity resin and size exclusion chromatography. The complex was blotted onto carbon gold grids and imaged using a Titan Krios microscope (Extended Data Fig. 2). The derived HU210/CB_1_/G_i1_ structure (Fig. 1a) has a global resolution of 2.9 Å and large overall similarity to published CB_1_/Gi_1–2_ complexes (Fig. 1b and Extended Data Table 2). The HU210-bound structure has the largest coverage of receptor residues among all reported active state CB_1_ structures^16–19,38,39^ (Extended Data Table 1). It covers 294 residues, which is four residue backbones and seven sidechains more than the second most complete structure^19^. The structure reveals more of the N-terminus wherein residues 100–105 form a b-hairpin-like bend with van der Waals contacts to residues F108^N-ter^, Q261^ECL2^, C264^ECL2^ and M371^ECL3^ (Fig. 1c). Intriguingly, we also observed structural changes in the G protein binding site, far away from the ligand binding site. The HU210 complex (with G_i1_) and CP55940 complex^19^ (with PAM and G_i2_) structures demonstrate distinct conformations of the modelled residues in the intracellular loop ICL3 (Fig. 1d). Altogether, the new HU210-bound structure expands our insights into the CB_1_/G_i1_ signaling complex and adds a high-resolution template for THC structure-activity studies.

HU210 shares binding site with the THC analogs AM841^16^ (Extended Data Fig. 3), CP55940^18,19^ (cryo-EM G_i1-2_ complexes), AM11542^16^ (crystal structure with a fusion protein, flavodoxin in ICL3) (Fig. 1e–f) bound to CB_1_ and AM12033-bound CB_2_^17^ (G_i1_ cryo-EM structure). Conformational clustering of the transmembrane helices of all active-state CB_1_ structures shows that ligands have a large impact on the structural similarity of receptors (Fig. 1g). Whereas the receptors first separate into crystal structures with fusion proteins (inducing an active-like conformation) and cryo-EM structures with G proteins, HU210-bound CB_1_ then groups closest to the cryo-EM complex with the closest analog of HU210, AM841 (Extended Data Table 1). These two ligands have a Tanimoto coefficient of 0.82 and their respective CB_1_/G_i1_ structure complexes have an RMSD value of 0.81 Å. Furthermore, the crystal and cryo-EM structures grouping the closest contain the same ligand, AM841 (Fig. 1g). This demonstrates a notably high conformational impact of the relatively small ligand on the overall CB_1_/G_i_ structure. Taken together, we extend the number of THC analogs with a CB_1–2_ structure to five and find that these bind with similar conformations in the same site and have a clear impact on the receptor conformation. These structures provide a strong support for structure-activity relationship analysis aiming to delineate common and unique receptor interactions (below).

### THC analogs exhibit diverse pharmacological activity

We selected 10 structurally diverse analogs of THC for investigation by docking, molecular dynamics and pharmacological assaying (Extended Data Fig. 1). This includes C1 phenolic hydroxyl removal (JWH133) and etherification (L759633), and C9 methyl replacement with carbonyl (Nabilone), hydroxy (CP55940) or hydroxymethyl (AM841, HU210 and HU243) groups. For the C3 alkyl tail, the analogs span propyl (THCv), butyl (JWH133), pentyl (THC and Cannabinol), heptyl (L759633, Nabilone, CP55940, HU243 and HU210) as well as heptyl substituted with isothiocyanate (AM841) or bromo (AM11542) groups. Of these 11 ligands, eight have an initial branching (1’,1’-dimethyl substitution) of the alkyl chain whereas THC, THCv and cannabinol lack this feature. Altogether, the selected 11 ligands allow for informative and efficient characterization of structure-function covering the major determinants^29–31^.

**Figure 2:**
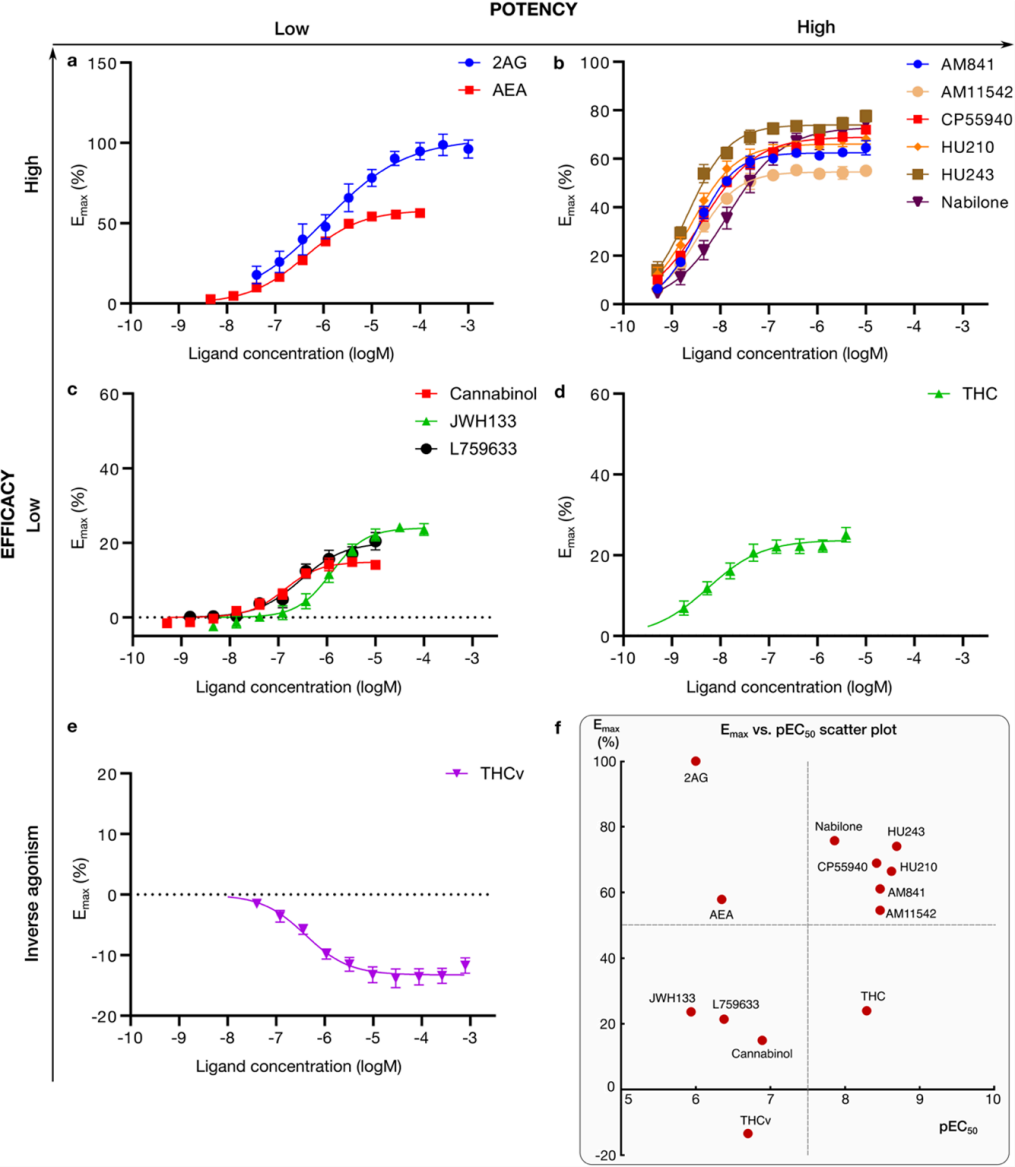
G protein recruitment of D^9^-THC analogs and endogenous agonists via CB_1_. Ligand concentration response curves for **a,** low-potency/high-efficacy agonists, **b,** high-potency/high-efficacy agonists, **c,** low-potency/low-efficacy agonists, **d,** high-potency/low-efficacy agonist, and **e,** inverse agonist, from mG_i_ recruitment. **f,** Scatter plot of ligand pEC_50_ (x-axis) and E_max_ (y-axis) in the mG_i_ experiments. HEK293-TR cells stably co-expressing a combination of either the CB_1_-NlucC and NES-venus-mGsi or NES-venus-b-arrestin were used. Recruitment data, expressed as a % of the maximal response produced by the endogenous cannabinoid 2AG, is plotted versus log concentration for the indicated ligands. Data were fitted to a four-parameter logistic equation; mean ± S.E.M. of at least 3 independent experiments.

**Figure 3:**
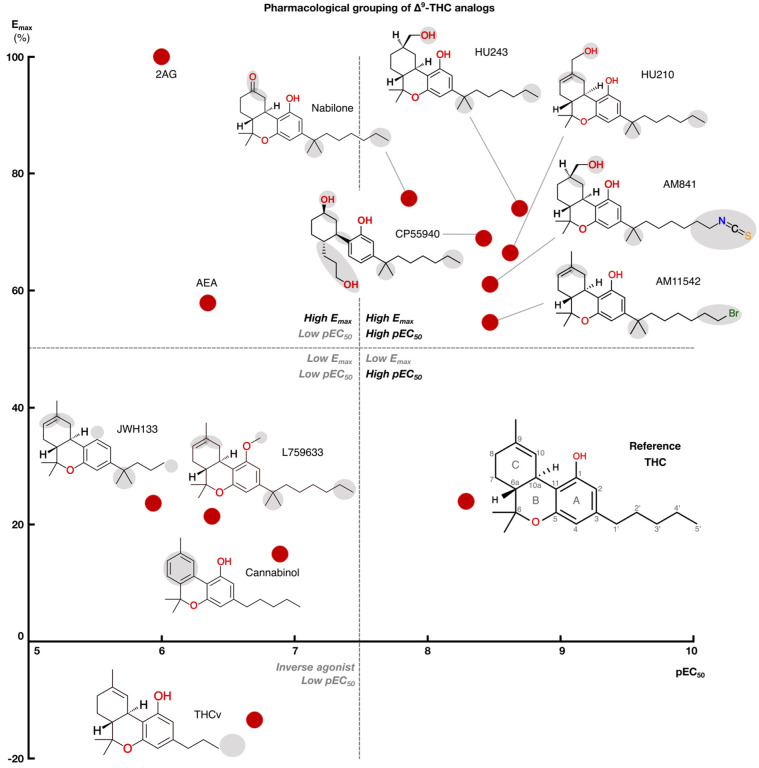
Structure-activity relationships of studied THC analogs. Scatter plot of ligand pEC_50_ (x-axis) and E_max_ (y-axis) in the mG_i_ experiments. Dotted lines divide the plot into quadrants of low/high pEC_50_/E_max_. For THC analogs, the 2D structure is shown next to the data points.

We profiled the recruitment of the primary transducer, G_i/o_ to CB_1_ for THC, the 10 analogs and the endocannabinoids 2-Arachidonoylglycerol (2AG) and N-arachidonoylethanolamine (AEA, Fig. 2 and Extended Data Fig. 1). We find that the tested ligands fall into five major groups by their activity: *i)* low-potency/high-efficacy (2AG and AEA), *ii)* high-potency/high-efficacy (AM841, AM11542, CP55940, HU210, HU243 and Nabilone), *iii)* low-potency/low-efficacy (Cannabinol, JWH133, L759633), *iv)* high-potency/low-efficacy (THC) and *v)* inverse agonist/antagonist (THCv) (Fig. 3). The endocannabinoid 2AG has the highest efficacy and was selected as the reference ligand for comparisons of efficacy (E_max_=100%).

### SAR of high-potency/high-efficacy THC analogs

**Figure 4:**
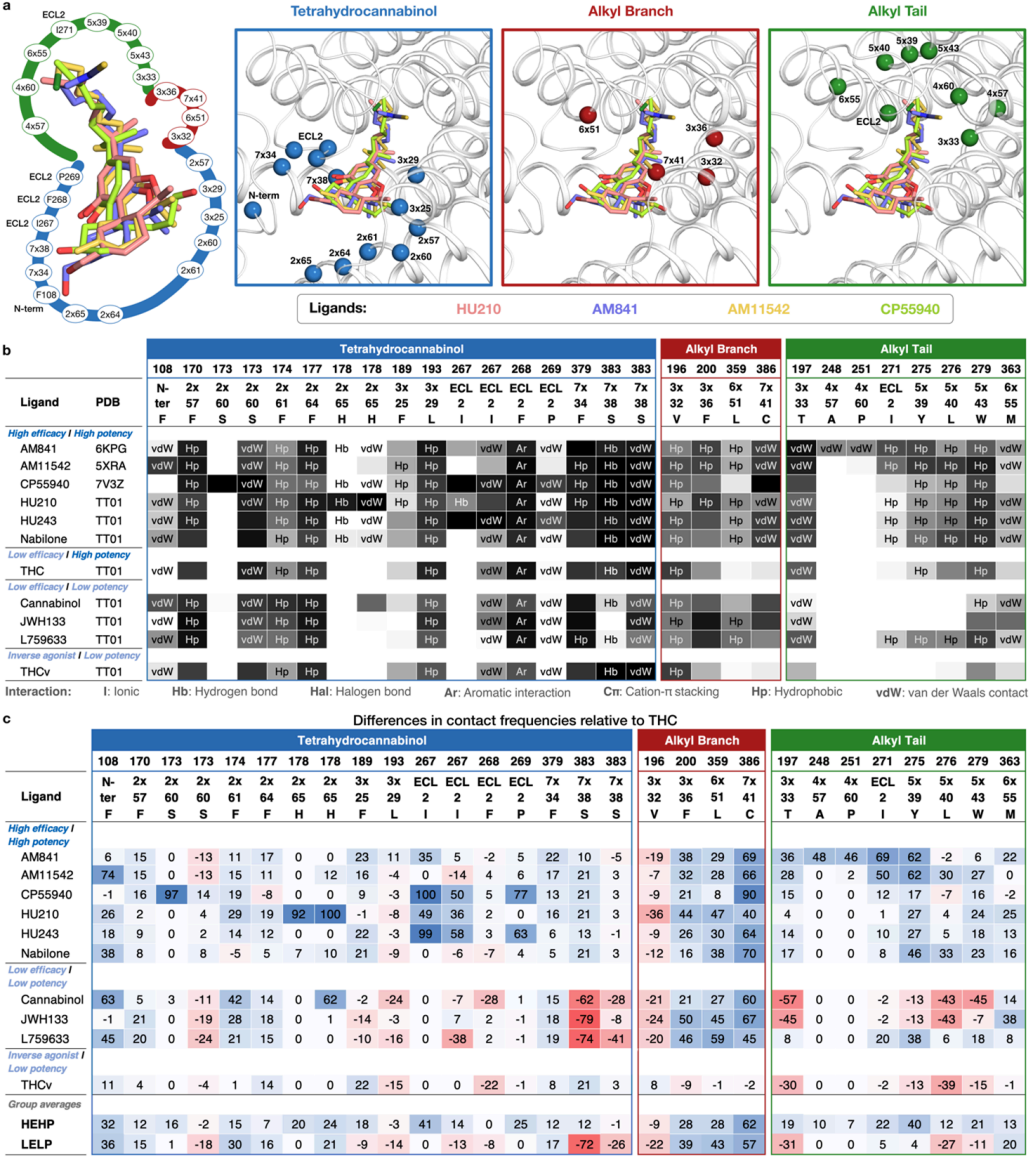
Spatiotemporal THC analog/CB_1_ interactions. a, Left: Spatial distribution of the 25 CB_1_ residues interacting with HU210 (pink), AM841^17^ (blue), AM11542^16^ (yellow), and CP55940^18^ (green). Right: The three subsites accommodating the tetrahydrocannabinol, alkyl branch and alkyl tail, respectively. Pocket residues are shown as C_a_ spheres and labelled with generic residue numbers facilitating comparison of structurally corresponding positions across receptors^75^. b, Ligand-receptor interaction fingerprints denoting interactions, type and CB_1_ generic residue positions. Interaction labels indicate receptor-ligand contacts in pre-MD starting structure/model (see Methods). Grayscale illustrates frequencies of interactions (%) throughout simulations. Five CB_1_ residue positions marked ‘N-ter’ and ‘ECL2’ lack a generic residue number because they are not structurally conserved across receptors. c, Differences in contact frequencies (in %) of THC analogs relative to THC (reference) at each receptor residue. Positive values (blue cells) indicate contact frequencies greater than those in THC, whereas negative values (red cells) indicate contact frequencies lesser than those in THC.

**Figure 5:**
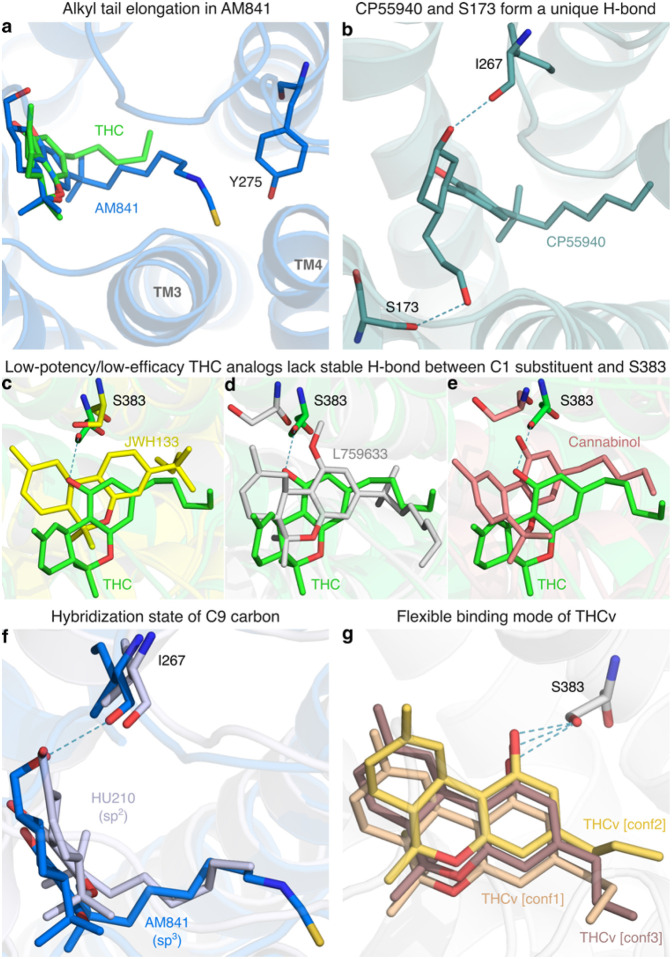
Structural determinants of ligand activity observed in MD simulations. Visual representations of ligand-receptor interactions from MD simulations that are key determinants of their structure-activity relationship. **a,** Conformation of the elongated alkyl tail of AM841 in comparison with THC. **b,** Binding mode of CP55940 depicting the unique hydrogen bond interaction between the 6a hydroxypropyl and the sidechain of S173^2×60^. **c-e,** Binding modes of low-potency/low-efficacy THC analogs (JWH133, L759633 and Cannabinol, respectively) in comparison with THC. **f,** Conformational differences in THC analogs having different hybridization states at C9 carbon of the C-ring; HU210 (sp^2^) and AM841 (sp^3^) are shown as representative analogs. **g,** Superposition of THCv conformations indicating binding flexibility.

All high-potency/high-efficacy THC analogs (AM841, AM11542, CP55940, HU210, HU243 and Nabilone) have an alkyl tail consisting of seven carbons - two more than the reference ligand THC. Through molecular dynamics (MD) simulations, we find that the high activity of the long alkyl chains may be attributed to favorable contacts to T197^3×33^, I271^ECL2^, Y275^5×39^ and W27 9^5×43^ that are 19, 22, 40 and 21% more frequent, respectively relative to THC (Fig. 4c). Conversely, a comparison with AM841, shows that a further extension of HU243 with an isothiocyanate moiety lowers ligand efficacy from 74% to 61% showing that a too long, or polar alkyl chain is not favorable. In MD simulations, AM841 positions the isothiocyanate substituent in an angled conformation between TM3 and TM4 where it induces a rotamer shift of Y275^5×39^ indicating a strained and/or higher-energy binding conformation (Fig. 5a). Like AM841, AM11542 also has a further elongated alkyl tail, being bromo-substituted, and its efficacy is lower than that of AM841.

The low efficacy of AM11542, compared to other analogs in this group, could also be attributed to the lack of a hydrogen bonding substituent at C9 at the opposite end of the ligand. Furthermore, Nabilone, which has the lowest potency in this group, has a carbonyl at C9 indicating that hydrogen bond donor capacity is essential for high activity. Consistently, the three agonists with the highest efficacy and potency have a hydroxyl (CP55940) or hydroxymethyl (AM841, HU210 and HU243) that can be hydrogen bond donors. In our MD simulations, CP55940’s C9 hydroxyl forms a very stable hydrogen bond to the backbone carbonyl of I267^ECL2^ (Fig. 5b). The hydroxymethyl-containing ligands share the hydrogen bond to I267^ECL2^ and have additional van der Waals contacts with H178^2×65^. Another difference at the C9 carbon is the hybridization state, which is sp^3^ for AM841, CP55940 and HU243, and sp^2^ for AM11542, HU210 and Nabilone. Our MD simulations show that ligands with sp^3^-hybridized C9 carbons, especially CP55940 and HU243, have a more stable hydrogen bond to the backbone carbonyl of I267^ECL2^ than observed for sp^2^-hybridized agonists (Fig. 5f). Nabilone instead has a unique, although low frequent, hydrogen bond between its C9 carbonyl and H178^2×65^.

All the ligands in this group have a 1’,1’-dimethyl substitution, which is important for activity. Uniquely, CP55940 lacks the B-ring and instead has a rotatable bond between the C10a and C11 atoms. As CP55940 groups close to the middle of the five other analogs it is possible that this flexibility does not affect activity. Alternatively, its effect could be masked by favorable interactions of the unique 6a hydroxypropyl. MD simulations show that this hydroxypropyl forms a very stable hydrogen bond to S173^2×60^ (Fig. 5b). Given this observation, and the major increase in conformational freedom at the center of the tricyclic core, the alternative explanation of a masked effect seems most plausible. Taken together, this presents a dynamic structural basis for high potency and efficacy that extends and agrees with previous structure-activities studies demonstrating the benefit of the seven-carbon alkyl chain^32,35^ and hydrogen bonding functionality (ideally donor) in position C9^31^.

### SAR of low-potency/low-efficacy THC analogs

Cannabinol is the single analog that groups closest to THC and it differs only by a single feature – an aromatic benzene in the C-ring. However, this difference alone leads to a 25-fold potency reduction (Extended Data Table 3). JWH133 has the lowest potency of all THC analogs and, like the inverse agonist THCv, has a shorter alkyl tail (one and two carbons fewer, respectively) than THC. JWH133 and L759633 both lack the 1-hydroxyl present in THC and the high-potency/high-efficacy analogs. JWH133, which has no 1-substituent, lacks an electron-donating group on the A-ring – weakening its aromatic interactions – and the hydrogen bond to S383^7×38^ in MD (Fig. 4c and Fig. 5c). Furthermore, in L759633, which has a 1-methoxy substituent, this hydrogen bond is 79% less frequent than in THC (Fig. 5d). Notably, although this hydroxyl group is preserved in Cannabinol this ligand also has an infrequent hydrogen bond – due to the conformational change induced by the planar C-ring (Fig. 4b and Fig. 5e). Hence, the 1-hydroxyl functionality and S383^7×38^ interaction are major contributors to potency. L759633 is structurally similar to HU210 differing only by the 1-substituent (methoxy vs. hydroxyl) and 9-substituent (methyl vs. hydroxymethyl). When considering the structure-activity relationships of all analogs, these two structural differences explain the low potency and efficacy, respectively of L759633.

### SAR of the inverse agonist THCv

Strikingly, THCv only differs from THC by a two-carbon reduction of the alkyl chain but has the opposite modality, inverse agonism and 39-fold lower potency. The large effect on potency is remarkable, given that the high-potency/high-efficacy analogs also differ by addition of two-carbons relative THC (albeit addition at a different site) but have similar potencies. The MD simulations show that the short propyl tail of THCv renders a part of the binding pocket vacant. Compared to THC, this leads to a loss of interactions in MD simulations to the residues Y275^5×39^, L27 6^5×40^ and I271^ECL2^. However, the MD analysis shows that a more profound characteristic of THCv is a much more dynamic overall binding mode (Fig. 5g). Throughout the simulations, THCv is anchored by a stable hydrogen bond between the central C1 hydroxyl and S383^7×38^ but its tricyclic ring moiety has less stable interactions with F268^ECL2^ and L193^3×29^ and the two distal ends are considerably more mobile than for THC. Altogether, the inverse agonism of THCv may be attributed in part to lost interactions of the tail but predominantly to the reduced stability of many more interactions across the scaffold.

### Low-potency/low-efficacy THC analogs exhibit G protein recruitment bias

To assess if THC analogs display different activity also in terms of biased signaling, we complemented the G_i1_ recruitment with arrestin recruitment experiments. By generating bias plots (Extended Data Fig. 4), we find that all ligands show a stronger recruitment of mG_i_ than arrestin. This indicates a general difference in the sensitivity of the two systems, whereas ligand bias can only be defined relative to a reference ligand^40^. We therefore calculated recruitment bias of tested ligands relative to two reference ligands, the endogenous ligand 2AG and THC. Interestingly, 2AG has low potency/high efficacy and THC has high potency/low efficacy (Fig. 3), but these ligands are nearly unbiased relative to each other because their opposite efficacy/potency relationships equal out when calculating log(E_max_/EC_50_) values. Specifically, their relative bias factor (fold-difference in log(Emax/EC50) across ligands and pathways) is a moderate 1.3 in both cases, for arrestin (THC) or G_i1_ (2AG).

The group of low-potency/low-efficacy THC analogs (Fig. 2c) show either very weak or no arrestin recruitment. For example, in the case of JWH133 its arrestin E_max_ was estimated to be 9.1 % of the 2AG response, while the response to L759633 was too low to fit to a curve and Cannabinol did not show any arrestin recruitment. Our observation that only low-potency/low-efficacy THC analogs display recruitment bias indicates that more chemical diversity would be required to achieve strong bias, while maintaining potency and efficacy.

### Structure-kinetics relationship

**Figure 6:**
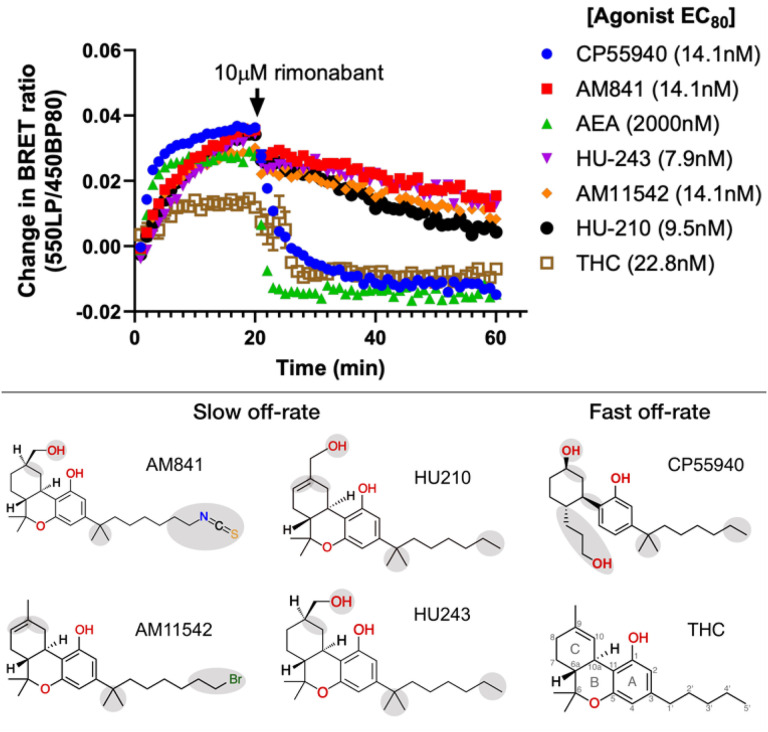
Structure-kinetics relationships of THC analogs studied for reversibility of agonist activity (mGj recruitment) upon addition of the CB_1_ antagonist rimonabant. The plot shows the change in basal BRET upon addition of agonist (0 min, EC_80_ concentration) and rimonabant (20 min, 10mM). Data from HEK293-TR cells stably expressing CB_1_-NlucC and NES-venus-mG_i_.

There have been contradictory results describing the binding of AM841 to CB_1_. Early studies based on washout^41,42^ experiments suggested that AM841 binding to CB_1_^41^ and CB_2_^42^ is irreversible and that this is due to the formation of a dithiocarbamate group between its isothiocyanate moiety and C355^6×47 43^. Additionally, irreversible AM841-CB_2_ binding was also suggested by a mass spectrometry study^43^. However, recent structures of AM841-bound CB_1_ provided evidence against this covalent interaction, as the ligand tail was not near C3 55^6×47^ in TM6 but instead occupied a hydrophobic pocket between TM3-TM5^16,17^. Furthermore, new washout experiments conducted with AM841 and analogs lacking the isothiocyanate moiety showed that although binding is “tight”^44^ or “wash-resistant”^16^, it is not irreversible or covalent. Notably, a study on functional effects in rhesus monkeys showed that THC does not share the pseudo-irreversible stimulation exhibited by HU210^45^. Altogether, these data point to different experimental effects with THC and its analogs. Therefore, we conducted a series of kinetics experiments designed to benchmark the apparent *k*_*o*ff_ values of THC and five analogs differing structurally in their tricyclic ring, alkyl branch and alkyl tail (Fig. 6, Extended Data Fig. 4 and Extended Data Table 4).

These experiments show the relative differences in ligand dissociation on mG_i_ binding following application of a saturating concentration of the CB1R specific inverse agonist rimonabant. We find that AM841, AM11542, HU210 and HU243 exhibit slow off-rates from the activated CB_1_ whereas THC, CP55940 and the endogenous CB1R agonist AEA instead show relatively fast off-rates. This is in agreement with previous studies demonstrating that the slow off-rate of these ligands is independent of an isothiocyanate moiety and covalent bond^16,44^, and that some ligands in this series have fast off-rates^45^. All four ligands with slow dissociation share the tricyclic ring, alkyl branch and alkyl tail with seven carbons. Given that CP55940 is the only ligand lacking the B-ring its difference in kinetics is likely caused by an increased conformational freedom or reduced bulk at the core scaffold. In MD simulations, CP55940 has an increased flexibility in this region destabilizing key hydrophobic interactions. For THC, the fast off-rate can instead be attributed to the unique shorter, five-carbon alkyl tail. As THC has a lower efficacy than its studied analogs it displays a smaller total drop in BRET ratio, whereas the steepest drop in binding is observed for the endocannabinoid AEA. Although its long aliphatic chain is similar to the C3 alkyl tail of THC analogs, AEA lacks the tricyclic scaffold fitting tightly into the binding pocket and making many hydrophobic interactions. Altogether, the kinetics experiments demonstrated that the tricyclic moiety, alkyl branch (1’,1’-dimethyl) and seven-carbon alkyl tail are essential features for THC analogs to bind to CB_1_ in a slow off-rate, “wash-resistant” manner.

## Discussion

Structural biology on GPCRs is approaching a throughput which allows comparison of complexes of close ligand analogs, especially when, like here they are complemented with manual docking or ligand substitution. Furthermore, high-performance computing has transformed molecular dynamics simulations, enabling the exploration of complex interactions involving multiple ligands bound to fully activated receptors, which in turn are bound to their cognate G proteins e.g.,^46,47^. A combination of these techniques allowed us to delineate detailed structure-activity relationships on the level of spatiotemporal ligand-receptor interactions. Moving beyond a solely static ligand-based analyses to one which encompasses receptor-effector complex explorations unlocks the potential for identifying and exploiting previously unexplored proximal binding sites, thus broadening the scope of therapeutic intervention and drug design strategies. Furthermore, it provides a deeper, target-complementarity rationale for favorable pharmacophore elements informing the optimal bioisosteric replacements. Exciting new prospects are currently opening for such rational, structure-based drug design from recent breakthroughs demonstrating highly accurate modeling of ligand-protein complexes using AlphaFold^48^ or RoseTTAFold All-Atom^49^. Finally, to attain the structural basis for design of better drugs through biased signaling, it would be highly desirable to determine complexes of differentially biased ligands bound to the same receptor and their respective preferred cognate transducer protein.

Our structure-activity relationship study covers diverse structural analogs of THC with modifications in key pharmacophore features and consistent pharmacological evaluation of potency, efficacy and signaling bias for G_i/o_ and b-arrestin. Our analysis adds to previous studies by plotting ligands by potency and efficacy (Fig. 3), allowing separation of ligands and determinants by both pharmacological properties. This is also the first comprehensive analysis of ligand-receptor binding modes. This took advantage of the new, high-resolution/-coverage CB_1_/G_i1_ structure providing a consistent receptor, while we could also preserve ligand conformations from recent experimental structures, where available^16–18^. The molecular dynamics simulations could explain differential activities not readily understood from ligand structures or static binding modes alone. For example, THCv only differs from THC by a two-carbon alkyl tail reduction but displays dramatic inverse agonism and low potency. Here, MD revealed a highly “wobbly” binding causing the receptor interactions to become unstable and infrequent. Another point in case for MD is that although preserving a C1 hydroxyl, Cannabinol – like the non-hydroxyl analogs JWH133 and L759633 – loses the hydrogen bond to S383^7×38^. This observation provides a structural basis contributing to Cannabinol’s relatively low affinity and potency at CB_1_ and to JWH133’s and L759633’s CB_2_ selectivity.

Our study is the first to report the relative rates of THC analog dissociation from an active CB_1_ receptor complex under consistent experimental conditions. These studied compounds vary in alkyl tail length (shorter in THC) and modifications (isothiocyanate in AM841 and bromine in AM11542), B-ring (open in CP55940) and C1’ alkyl branch (missing in THC). Furthermore, we included the endocannabinoid AEA as an additional, non-THC-analog reference ligand. Our results demonstrate that the length of the alkyl tail and completeness of the tricyclic ring moiety are structural determinants for dissociation kinetics from the active form of the receptor. This corroborates a recent study that performed washout experiments of 14 compounds similar to AM841^44^ – all of which had a heptyl or octyl tail and displayed wash-resistant binding. The tight binding which results in slow dissociation, is likely due to the ligands’ fit in the binding pocket and molecular interactions, the majority of which are hydrophobic contributing to the free energy of binding. The early belief that AM841 binds irreversibly due to a covalent bond was based on the observation that mutations of C3 55^6×47^ to serine, alanine and leucine significantly increased radioligand binding relative to wildtype CB_1_ in the presence of AM841^41^. This has now been disproven by AM841-CB_1_ structure complexes^16,17^ and recent washout studies^16,44^. Of note, C3 55^6×47^ is part of a highly conserved C3 55^6×47^WxP motif in TM6 involved in ligand binding and activation of class A GPCRs^50^. Hence, the most plausible explanation of the mutagenesis effects is through weakening of ligand binding and receptor activation.

The THC analogs HU210 and CP55940 have been previously reported to be biased towards the G_i/o_ family (cAMP inhibition) over phosphorylation of extracellular signal-regulated kinase 1/2 (pERK1/2)^51^. However, pERK1/2 is a downstream protein with activity that is dependent on both G proteins and arrestins. Therefore, its bias cannot be delineated directly at the level of the receptor-binding transducers. Our experiments separate these pathways and show that only low-potency/low-efficacy THC analogs have a strong bias towards G protein over arrestin recruitment, which is weak or abrogated (Extended Data Fig. 4 and Extended Data Table 3). This indicates that that more chemical diversity would be required to achieve strong bias, while maintaining potency and efficacy. Several compound classes activating CB_1_ have already been found to exhibit biased signaling, including endocannabinoids, phytocannabinoids, synthetic cannabinoids, indoles and biphenylureas^13^. Furthermore, in our experiments, THC displayed a slight preference (bias factor 1.3) for arrestin over G_i1_ compared to the endogenous agonist 2AG. All biased signaling experiments are heavily system-dependent and these experiments, performed on transducers directly binding to the receptor, often do not translate to *in vivo* settings^40^. For example, both THC and 2AG have been shown to be arrestin-selective over G_i/o_ in a cell model of striatal neurons^52^. Together, this warrants many more biased signaling studies covering different CB_1_ ligand scaffolds across the transducer, downstream pathway and *in vivo* levels. For example, to investigate if biased ligands can be identified that induce therapeutic effects, such as analgesia, without common side effects e.g., hypothermia, catalepsy, and hypolocomotion^53^.

Taken together, the HU210/CB_1_/G_i1_ cryo-EM structure, pharmacological profiling, ligand-receptor docking and molecular dynamics simulations could link functional groups of THC ligands to spatiotemporal interactions determining efficacy, potency, biased signaling and kinetics. This may advance our understanding of fundamental receptor function and support the further development of THC and its analogs as therapeutics.

## Methods

### Protein expression

Human cannabinoid receptor 1 (CB1) residue 2–472 was fused with a N-terminal hemagglutinin (HA) signal peptide followed by a Flag tag and a short Gly-Ser linker. At its C-terminus, the receptor was fused to a 10-histidine purification tag. The DNA sequence was codon optimized for expression in insect cells and inserted into the pFastBac1 vector using custom DNA synthesis (Genscript) for Bac-to-Bac virus expression. A dominant negative mutant of human GNAi1 (G203A/A326S)^37,54^ was also inserted into PfastBacl. Viruses co-expressing human GNB1 and GNG2 together, as well as Ric8A were kindly provided as a gift by Daniel Hilger. The CB1 receptor Gi protein complex was formed by co-expression in *Spodoptera frugiperda* (S*f*9) insect cells. Cells were infected at 2 mio/ml cells with vius at a ratio between receptor, GNAil, GNB1/GNG2, and Ric8A at (8:5:2:1). Cells were harvested by centrifugation after 48 hours and frozen before subsequent purification.

A Fab fragment (scFv16) construct essentially identical to the construct reported by ^55^ was expressed using pFastBacl in BTI-Tn-5B1–4 (High Five/Hi5) cells. Cells were infected at 2 mio/ml cells and harvested after 48 hours. Cells were removed by centrifugation and the supernatant containing the secreted scFv16 protein was frozen with 10% glycerol.

### ScFv16 purification

ScFv16 was purified using HisPur^™^ Ni-NTA resin (Thermofisher sci. Cat. No. 88221). In short, initially impurities were removed by precipitation by adding 30 mM Tris pH 8.0, 1 mM NiSO_4_ and 5 mM CaCl_2_ for 1 hour at room temperature followed by centrifugation at 18,000 rpm for 20 min. The supernatant was filtered through a 0.22 mM filter and loaded onto Ni-NTA resin over night at room temperature. The resin was then washed in wash buffers: Wash buffer 1: 20 mM Hepes pH 7.4, 500 mM NaCl and 10 mM imidazole, wash buffer 2: 20 mM Hepes pH 7.4, 100 mM NaCl and 10 mM imidazole and wash buffer 3: 20 mM Hepes pH 7.4, 100 mM NaCl and 30 mM imidazole. The protein was eluted with a buffer containing 20 mM Hepes pH 7.4, 100 mM NaCl and 300 mM imidazole. The histidine tag of the protein was removed by digesting with 3C protease for 3 days at 4 °C. ScFv16 was concentrated to ~1.5 mg/ml and frozen with 20% glycerol.

### Complex formation and purification

The cell pellet from 1 L of media containing the CB1 Gi protein complex was lysed at room temperature for 1 hour with stirring. The lysis buffer consisted of: 20 mM Hepes pH 7.4, 50 mM NaCl, 4 mM MgCl_2_, 2 mM HU-210 (Tocris Cat. No. 0966), ~10 mg/ml ScFv16, 0.025 units/ml Apyrase (NEB Cat. No. M0398S) and Roche EDTA free protease inhibitors (Sigma Cat. No. REF05056489001). The lysed cells were harvested by centrifugation for 20 min at 11,000 rpm. The complex was then solubilized using a 40 ml dounce tissue grinder with 30 strokes and a solubilization buffer containing 20 mM Hepes pH 7.4, 100 mM NaCl, 4 mM MgCl_2_, 1 mM HU-210, ~20 mg/ml ScFv16, 0.05 units/ml Apyrase (NEB Cat. No. M0398S), 15% glycerol, 0.5% LMNG (Anatrace Cat. No. NG310), 0.03% Cholesteryl hemisuccinate (Sigma Cat. No. C6512–25G) and Roche EDTA free protease inhibitors. The sample was mixed for 2 hours at 4 °C. Non solubilized material was removed by centrifugation for 40 min at 14.000 rpm. 5 mM CaCl_2_ and 0.75 ml M1 anti flag resin added to the supernatant. The slurry was gently mixed for 1 hour at 4 °C and the beads with bound complex were collected by centrifugation for 5 min at 1000 g and transferred to a 1.5 ml filter column (Bio-Rad Cat. No. #7311550EDU). The resin was washed with purification buffer containing 20 mM Hepes pH 7.4, 100 mM NaCI, 5 mM CaCl_2_, 2 mM MgCl_2_, 500 nM HU-210, 0.01% LMNG and 0.006% Cholesteryl hemisuccinate . The protein was eluted in purification buffer supplemented with 10 mM EDTA and 200 mg/ml Flag peptide (Sigma Cat. F3290–4MG). The eluted protein was supplemented with an additional 1 mM HU-210 and concentrated to ~50 ml using 100 kDa spin concentrators (Merck cat. nr. Z614092) at 1,000 g. Subsequently, the complex was frozen in liquid nitrogen. Immediately before preparation of grids for cryo-EM, the sample was run on a Superose 6 Increase 3.2/300 column on a Akta system using purification buffer for elution at 0.05 ml/min.

### Cryo-EM

Prior to sample application the cryo-EM Quantifoil R 1/1 C 300 grids were activated using a Gloqube Plus glow discharger at 15 mA for 45 sec. All grids were prepared using a Leica EM2 plunge freezer. 3 ml of complex at ~0.85 mg/ml was loaded using a total blotting time of 6–8 sec.

### Cryo-EM data collection

Data collection was performed on a Titan Krios G3i (Thermo Fisher Scientific) with a K3/BioQuantuum detector/energy filter setup (Gatan). Magnification was set at 130kx, resulting in a physical pixel size of 0.647 Å/px. Automated data collection was done using EPU (Thermo Fisher Scientific) set to collect in super resolution with 2x binning to the physical pixel size, with gain correction on the fly. AFIS was used for faster data collection speed with 1 exposure per hole for 11260 movies total. Defocus targets were set from −0.6 to −1.8 μm in steps of 0.2 μm, with autofocus after distance at 6 μm. A 50 μm C2 aperture and no objective aperture was used. The energy filter was tuned and set to a slit width of 20 eV with auto centering of the ZLP every hour. An exposure time of 1.50 s in 56 frames for 60.6 e/Å^2^ total dose was employed.

### Cryo-EM data processing

CryoSPARC Live (Structura Biotechnology, ^56^) was used to monitor data quality during the data collection and provided an initial 3D volume. The data was pre-processed in Relion 3.1^57^ followed by particle picking using crYOLO^58^ resulting in 603k particles. The particle stack was cleaned by iterating between jobs of 3D Classification and 3D Auto-refine using Sidesplitter^59^, ending with a polished stack of 187k particles. The stack was imported to CryoSPARC and subjected to Non-uniform 3D refinement, followed by a Local Refine job with a mask around the TM region resulting in a 2.9 Å overall resolution.

### HU210/CB_1_/G_i1_ structure model building

The published structure of CB1 bound to the ligand AM841 (PDB: 6KPG)^17^ was used as the starting model for our refinement. The starting model for the alpha helical domain of the G_i1_ subunit was retrieved from the crystal structure of the scFv16 bound G_i1_ heterotrimer (PDB: 6CRK)^60^. Model building and refinement were done using the Coot^61^ and Phenix^62^ software packages.

### THC analog – CB_1_ structure/model preparation

The HU210/CB_1_/G_i1_ structure was prepared for THC analog docking and molecular dynamics simulations with THC and analogs. We removed ScFv16, reverted the two Ga_i1_ mutations (G203A and A326S) to wildtype and modeled-in the missing ICL3 (residues 316–334) of CB1 using MODELLER^63^. The structure was prepared using the Protein Preparation Wizard tool^64^ and OPLS3e force field^65^ in Schrodinger^66^. For, AM841^17^, AM11542^16^ and CP55940^18^ conformations were taken from their respective CB_1_ structure complexes (the latter two lacking a G protein) and superposed to HU210 to generate CB_1_/G_i1_ complexes. For the remaining THC analogs, CB_1_/G_i1_ complexes were constructed by using the Maestro 3D Builder Tool^66^ to edit HU210 in our CB_1_/G_i1_ structure and perform energy minimization.

### Molecular dynamics simulations

Protonation states of ionizable residues were assigned using Epik^66,67^ using a pH value of 7.0 as reference. Restrained minimization was performed using the OPLS3e force field^65^. A membrane was built and positioned around the receptor with guidance from the PPM 2.0 Web Server of the Orientations of Proteins in Membranes (OPM) database^68^. An orthorhombic box shape with 11 Å buffer distance in the x, y and z directions was used to build a solvent box around the membrane-bound agonist-receptor-transducer complexes. All atom 1-palmitoyl-2-oleoylphosphatidylcholine bilayer (POPC) and TIP3P water were used for the lipid and water models, respectively. A salt concentration of 0.15 M was set, and all the systems were neutralised using sodium and chloride ions.

All MD simulations were performed using the Desmond Molecular Dynamics System^69^ with the OPLS3e force field^65^ and full particle mesh Ewald electrostatics^70^. The systems were gradually heated to 300 K in the NVT ensemble and allowed to equilibrate for 50 ns. Production MD simulations at constant temperature (300 K) and pressure (1 atm) were performed for 1000 ns for each system. This resulted in a total simulation time of 11 μs from all the simulations. Analysis of MD trajectories to calculate frequencies of receptor-ligand, intra-receptor, and receptor-transducer contacts was done using the GetContacts MD analysis package^71^.

### Cell culture and molecular biology reagents

The HEKT-REx^™^-293 cell line (Cat. No. R71007) was purchased from Invitrogen (CA, USA). T75 mammalian cell culture flasks were purchased from Fisher Scientific (Loughborough, UK). Cell culture reagents from Sigma Aldrich (St. Louis, MO, USA) include Dulbecco’s Modified Eagle’s Medium (DMEM) – high glucose (Cat. No. D6429), Dulbecco’s Phosphate Buffered Saline (D-PBS, Cat. No. D8537), Hank’s Buffered Saline Solution (HBSS, Cat. No. H8264), 4-(2-hydroxyethyl)-1-piperazineethanesulfonic acid) sodium salt (HEPES, Cat. No. RDD035–100G), bovine serum albumin (FBS, Cat. No. F7524), trypsin/EDTA solution 100mL (Cat. No. R001100). Reagents purchased from Gibco^™^ (MA, USA) included Blasticidin^™^ Selection Reagent HCl 10mg/mL (Cat. No. 12172530), Zeocin^™^ Selection Reagent 100mg/mL (cat. No. R25005), Geneticin^™^ Selective Antibiotic (G-418 Sulfate) 50mg/mL (Cat. No. 10131035). Reagents from Corning^®^ (Corning, NY, USA) include Corning^®^ 100mL Cellstripper^™^, liquid (Cat. No. 25–056-Cl). Polyethylenimine (PEI, Cat. No. 23966–1) was obtained from Polysciences Inc (PA, USA). 96-well cell culture plates (Cat. No. 655098) were purchased from Greiner Bio-One (Stonehouse, UK). The expression vectors pcDNA^™^4/TO was obtained from ThermoFisher Scientific and pcDNA^™^ 3.1 from Invitrogen^™^.

### Compounds

Reference ligands were obtained from Bio-Techne^®^ Tocris (Abingdon, Oxfordshire, UK) include HU210 or ((6a*R*)-*frans*-3-(1,1-Dimethylheptyl)-6a,7,10,10a-tetrahydro-1-hydroxy-6,6-dimethyl-6*H*-dibenzo[*b,d*]pyran-9-methanol) (Cat. No. 0966), anandamide (AEA) or (*N*-(2-Hydroxyethyl)-5*Z*,8Z,11Z,14*Z*-eicosatetraenamide (Cat. No. 1339), 2-Arachidoylglycerol (2AG) or (5*Z*,8Z,11Z,14*Z*)-5,8,11,14-Eicosatetraenoic acid, 2-hydroxy-1-(hydroxymethyl)ethyl ester (Cat. No. 1298), and Rimonabant (SR-141716A) or (*N*-(Piperidin-1-yl)-5-(4-chlorophenyl)-1-(2,4-dichlorophenyl)-4-methyl-1*H*-pyrazole-3-carboxamide hydrochloride) (Cat. No. 0923), (−) Cannabinol or 2-[(1R,6R)-3-Methyl-6-(1-methylethenyl)-2-cyclohexen-1-yl]-5-pentyl-1,3-benzenediol (Cat. No. 1570), JWH133 or (6aR,10aR)-3-(1,1-Dimethylbutyl)-6a,7,10,10a-tetrahydro-6,6,9-trimethyl-6H-dibenzo[b,d]pyran (Cat. No. 1343/10). CP55940 or (5-(1,1-Dimethylheptyl)-2-[5-hydroxy-2-(3-hydroxypropyl)cyclohexyl]phenol) (Cat. No. C1112), Nabilone (Cat. No. N3785), Tetrahydrocannabivarin (THCv) (Cat. No. T-094) and Δ9-Tetrahydrocannabinol solution (THC, Cat. No. T2386) was obtained from Merck. L759633 (Cat. No. CAY10009280–1 mg) was obtained from Cambridge Biosciences.

### Cell culture

Cultured cells were maintained in a humidified incubator at 37 °C and 5% CO_2_ in Dulbecco’s modified Eagle’s medium (DMEM) (Sigma-Aldrich) containing 10% foetal bovine serum (FBS). Stable cell lines expressing both the CB_1_ plus venus-mGsi, and the CB_1_ plus venus-β-arrestin2, were created in T75 flasks, using a 3:1 ratio of polyethylenimine (PEI): DNA. 24 hours post transfection cells were split into T175 flasks and maintained as stable cell lines. Antibiotics including blasticidin (5 μg/mL) and Zeocin^™^ (20 Mg/mL) were introduced as selection agents to create stable cell lines expressing the pcDNA^™^4/TO a mammalian expression vector which encodes the appropriate CB_1_ sequence (ThermoFisher Scientific, UK). Whilst Geneticin^®^ (G-418 sulphate, 200 μg/mL) was used to select for cells containing the pcDNA^™^ 3.1 mammalian expression vector (Invitrogen^™^), encoding venus-mGsi and venus-β-arrestin2. A mixed population of stable cells was eventually produced with cells having resistance to the selection agents employed.

### Mini-G protein and β-arrestin recruitment assays in CB_1_-expressing cells.

CB_1_ coupling to G proteins was assessed using a fluorescent G protein surrogate, venus-mini-Gsi1 (vmGsi) protein^72^. The venus-mGsi subunit is essentially a chimeric protein consisting of C-terminal Gi1 residues grafted onto venus-mGs, and originally engineered from the native Gsa protein. The venus-mGsi is ideal for studying receptor activation of the CB_1_, as unlike its wild type Gai1 counterpart, the resulting active-receptor complex formed is stable and resistant to nucleotide exchange meaning that active state signalling is maintained if the agonist is present.

HEK293TR-CB_1_-nLuc cells expressing fluorescently labelled miniG or β-arrestin protein were maintained in a humidified environment at 37 °C and 5% CO_2_ in Dulbecco’s modified Eagle’s medium (DMEM) with 10% fetal bovine serum (FBS) containing blasticidin (5 mg/ml), Zeocin (20 mg/ml) and G418 (0.2mg/mL) and used to assess compound stimulated β-arrestin recruitment to the human CB_1_. Cultured cells were harvested upon reaching 70% confluency and plated at a seeding density of 50,000 cells per well in poly-D-lysine (5 mg/mL) coated clear-bottomed 96-well cell culture plates. The cells were grown for 48h until they reached confluency and then stimulated with (1 μg/mL tetracycline) for a further 48h. Media was aspirated, and the cells were washed in 100 μL/well PBS, then 90 μL/well assay buffer (HBSS, 0.5% BSA, 5 mM HEPES) with 10 μM furimazine was applied to each well. A white back seal was applied to plates, which was then incubated for 15 minutes at 37°C to allow the furimazine to enter the cells. Assay plates were then transferred to the PHERAstar FSX set to a temperature of 37°C, and three BRET cycles were run to collect an initial baseline reading, after which 10 μL of compounds diluted in assay buffer was added to the plate, and the plate was read at 1-minute intervals for 30 minutes. Compounds were serially diluted in DMSO, before a 1/10 dilution in assay buffer and a further 1/10 dilution on addition to the assay plate. Buffer containing 10% DMSO (1% final) served as the vehicle control with all responses normalised to the maximal response produced by 2AG. mini-G protein reversal experiments were performed essentially as described above, by applying a concentration of agonist producing 80% of its own maximum response (EC_80_) to cells in a 96-well cell culture plate, followed by the addition of an excess of rimonabant (10 mM) or vehicle, with the resulting BRET signals monitored for up to 90min.

### Signal detection and data analysis

Raw experimental mG_i_ and β-arrestin2 recruitment data was collected at 1 min intervals on the BMG PHERAstar FSX (BMG Labtech, Offenburg, Germany), and processed using MARS data analysis software (BMG Labtech), as the ratio of BRET 1 (535–30LP/475–30BP). This data was then exported in Microsoft Excel and transferred to GraphPad PRISM 9.2 (GraphPad Software, San Diego, U.S.A.). A kinetic analysis of compound induced response measured over time was completed by plotting the resulting reported BRET ratios. Characterisation of agonist CBR responses was achieved by selecting the concentration-response data at a fixed time point and one producing the maximal observable responses to the ligands under test. Concentration response data was then normalized to the reference ligand 2AG. Individual concentration response data were fitted to sigmoidal (variable slope) curves using a “four parameter logistic equation”:

(1) $$\text{Y}=\text{Bottom}+(\text{Top}-\text{Bottom})/(1+10^{\left({\text{LogEC}}_{50}-\text{X}\right)*\text{Hillslope}})$$

Where Bottom and Top are the plateaus of the agonist and inverse agonist concentration response curves. LogEC_50_ is the concentration of agonist/inverse agonist that gives a half-maximal effect, and the Hillslope is the unitless slope factor. Individual agonist EC_50_ and E_max_ values are reported as the Mean ± SEM, from the number (n) of individual experiment indicated.

All normalized data of the individual concentration points from each individual experiments were pooled, and bias plots were constructed by means of a ‘centered second-order polynomial’ fitting of the normalized and pooled data obtained for the individual concentration points of the mini-Gi (x-coordinate) and b-arrestin2 (y-coordinate) assay formats.

The rates of mini-Gi protein reversal were estimated for each agonist in Prism 9.0 using the following equation which describes a ‘one phase exponential decay’:

(2) $$\text{Y}=\text{Span}*{\text{exp}}^{\left(-k\text{off}*\text{X}\right)}+\text{Plateau}$$

Individual agonist *k*_off_ values are reported in tables as the Mean ± SEM, from the number (n) of individual experiment indicated.

### Compound synthesis

*(6aR,9R,10aR)-9-(hydroxymethyl)-3-(8-isothiocyanato-2-methyloctan-2-yl)-6,6-dimethyl-6a,7,8,9,10,10a-hexahydro-6H-benzo[c]chromen-1-ol (AM841)*. To a stirred solution of (6aR,9R,10aR)-3-(8-azido-2-methyloctan-2-yl)-9-(hydroxymethyl)-6,6-dimethyl-6a,7,8,9,10,10a-hexahydro-6H-benzo[c]chromen-1-ol (56 mg, 0.13 mmol) in anhydrous tetrahydrofuran (2.6 mL) at room temperature under an argon atmosphere were added triphenylphosphine (170 mg, 0.65 mmol) followed by carbon disulfide (230 uL, 3.9 mmol) and the mixture was stirred at that temperature for 24 hours. Upon completion, the volatiles were evaporated under reduced pressure and the residue was purified by flash column chromatography (silica gel; 25% ethyl acetate in hexanes as eluent) to afford 49 mg of AM841. Physical, analytical, and spectroscopic data were identical to those we reported earlier^16^.

*(6aR,10aR)-3-(8-bromo-2-methyloctan-2-yl)-6,6,9-trimethyl-6a,7,10,10a-tetrahydro-6H-benzo[c]chromen-1-ol (AM11542)*. To a mixture of 5-(8-bromo-2-methyloctan-2-yl)benzene-1,3-diol (124 mg, 0.39 mmol) and p-toluenesulfonic acid (14 mg, 0.08 mmol) in anhydrous methylene chloride (3 mL) at 0°C under an argon atmosphere was added a solution of (4R)-1-methyl-4-(prop-1-en-2-yl)cyclohex-2-en-1-ol (71 mg, 47 mmol) in anhydrous methylene chloride (1 mL) and the resulting mixture was stirred at that temperature for 40 minutes. Upon completion, the reaction was quenched by saturated aqueous sodium bicarbonate and the organic layer was dried over sodium sulphate and evaporated. The residue was purified by flash column chromatography (silica gel; 5% diethyl ether in hexanes) to afford 127 mg of (1’R,2’R)-4-(8-bromo-2-methyloctan-2-yl)-5’-methyl-2’-(prop-1-en-2-yl)-1’,2’,3’,6’-tetrahydro-[1,1’-biphenyl]-2,6-diol. The product (122 mg, 0.27 mmol) was dissolved in anhydrous methylene chloride (8 mL) under an argon atmosphere and cooled to 0°C. Boron trifluoride etherate (160 uL, 1.3 mmol) was added and the mixture was stirred for 30 minutes at 0°C followed by 6 hours at ambient temperature. The reaction was quenched by saturated aqueous sodium bicarbonate and the organic layer was dried over sodium sulphate and evaporated. The residue was purified by flash column chromatography (silica gel; 5% ethyl acetate in hexanes) to afford 105 mg of AM11542. Physical, analytical, and spectroscopic data were identical to those we reported earlier^73^.

## Data Availability

The HU210/CB_1_/G_i1_ cryo-EM structure and density are available in the Protein Data Bank under accession code PDB 9ERX.
